# The Role of Informative and Ambiguous Feedback in Avoidance Behavior: Empirical and Computational Findings

**DOI:** 10.1371/journal.pone.0144083

**Published:** 2015-12-02

**Authors:** Ahmed A. Moustafa, Jony Sheynin, Catherine E. Myers

**Affiliations:** 1 Department of Veterans Affairs, New Jersey Health Care System, East Orange, NJ, United States of America; 2 School of Social Sciences and Psychology & Marcs Institute for Brain and Behaviour, University of Western Sydney, Sydney, New South Wales, Australia; 3 Veterans Affairs Ann Arbor Healthcare System, Ann Arbor, MI, United States of America; 4 Department of Psychiatry, University of Michigan, Ann Arbor, MI, United States of America; 5 Department of Pharmacology, Physiology & Neuroscience, Rutgers-New Jersey Medical School, Newark, NJ, United States of America; 6 Department of Psychology, Rutgers University-Newark, Newark, NJ, United States of America; Centre national de la recherche scientifique, FRANCE

## Abstract

Avoidance behavior is a critical component of many psychiatric disorders, and as such, it is important to understand how avoidance behavior arises, and whether it can be modified. In this study, we used empirical and computational methods to assess the role of informational feedback and ambiguous outcome in avoidance behavior. We adapted a computer-based probabilistic classification learning task, which includes positive, negative and no-feedback outcomes; the latter outcome is ambiguous as it might signal either a successful outcome (missed punishment) or a failure (missed reward). Prior work with this task suggested that most healthy subjects viewed the no-feedback outcome as strongly positive. Interestingly, in a later version of the classification task, when healthy subjects were allowed to opt out of (i.e. avoid) responding, some subjects (“avoiders”) reliably avoided trials where there was a risk of punishment, but other subjects (“non-avoiders”) never made any avoidance responses at all. One possible interpretation is that the “non-avoiders” valued the no-feedback outcome so positively on punishment-based trials that they had little incentive to avoid. Another possible interpretation is that the outcome of an avoided trial is unspecified and that lack of information is aversive, decreasing subjects’ tendency to avoid. To examine these ideas, we here tested healthy young adults on versions of the task where avoidance responses either did or did not generate informational feedback about the optimal response. Results showed that provision of informational feedback decreased avoidance responses and also decreased categorization performance, without significantly affecting the percentage of subjects classified as “avoiders.” To better understand these results, we used a modified Q-learning model to fit individual subject data. Simulation results suggest that subjects in the feedback condition adjusted their behavior faster following better-than-expected outcomes, compared to subjects in the no-feedback condition. Additionally, in both task conditions, “avoiders” adjusted their behavior faster following worse-than-expected outcomes, and treated the ambiguous no-feedback outcome as less rewarding, compared to non-avoiders. Together, results shed light on the important role of ambiguous and informative feedback in avoidance behavior.

## Introduction

Avoidance refers to action that is taken to prevent the occurrence of aversive or undesired events. While avoidance is an adaptive response to danger, exaggerated or pathological avoidance is a core symptom in many clinical disorders, including posttraumatic stress disorder (PTSD) [1, 2], obsessive-compulsive disorder [3], and addiction [4]. Understanding avoidance behavior in healthy populations, as well as how to modify or decrease avoidance behavior, will then have implications for the study and treatment of clinical patients with avoidance symptoms.

The study of avoidance in a laboratory setting has been investigated by training subjects to avoid aversive stimuli such as electric shocks [5, 6] or point loss in computer-based games [4, 7, 8]. For example, in previous work, we and others have used computer-based probabilistic classification tasks in which subjects learn from both rewarding and punishing feedback. In the "standard" version of the task, there are positive (+25 points), negative (-25 points) and ambiguous (no feedback) outcomes [9].

Previous work with this task suggested that the relative rates of reward- and punishment-based learning vary widely in different neurological and psychiatric groups. Thus, for example, veterans with symptoms of post-traumatic stress disorder (PTSD), which includes avoidance as a defining symptom, performed better on the task, particularly on reward-based trials, compared to peers with few/no PTSD symptoms [10]. A similar pattern was observed in putatively healthy young adults (college students), those with the personality trait of behavioral inhibition, a tendency to withdraw from or avoid novel stimuli [8]. Conversely, *de novo* (never-medicated) patients with Parkinson’s disease (PD) showed deficits on reward-based learning but normal punishment-based learning; dopaminergic treatment remediated the punishment-based learning but introduced deficits on reward-based learning [9, 11]. Interestingly, the “unmedicated PD” pattern of selective deficiency in reward-based learning was also observed among asymptomatic carriers of a rare duplication in the *SNCA* gene which causes one form of PD [12]. These latter data are consistent with the idea that nigrostriatal dopamine systems, which are damaged in PD, are important for signaling reward, or predicted reward, in the brain [13, 14].

To interpret such results, we and others have used computational models to evaluate individual differences in how subjects approach the task [15–19]. Specifically, reinforcement-learning (RL) models allow simulation of each subject’s pattern of trial-by-trial behavior, and estimation of parameters governing that behavior, such as each subject’s learning rate and the subjective value of ambiguous feedback. Between-subject differences in estimated parameter values can help explain between-subject differences behavioral performance. We applied such an RL model to the veteran data, and found that those with more severe PTSD symptoms had lower estimated values of the no-feedback outcome, that is, they treated ambiguous outcome as less rewarding, compared to peers with few/no PTSD symptoms [10]. This in turn suggests that the interpretation of neutral, ambiguous outcome may be an important component in understanding avoidance, and begs further study of how neutral, ambiguous reinforcement is processed.

Another way in which no-feedback outcomes can be provided is to allow the subject, on each trial, the option of simply avoiding (i.e., "skipping") the trial rather than making a classification response. In this case, the task simply moves on to the next trial, and there is no positive or negative feedback. Since the task is probabilistic, theoretically optimal behavior is therefore to avoid all punishment-based trials (thereby avoiding any risk of punishment) while attempting categorization responses on all reward trials (where there is no risk of punishment). Sheynin et al. [8] indeed found that healthy young adults tended to make avoidance responses on very few reward-based trials, while number of avoidance responses in punishment-based trials tended to increase over the experiment; however, over a third of subjects did not make any avoidance responses during the experiment.

These results therefore raise the question of why so many putatively healthy subjects do not make avoidance responses, even on trials where they cannot receive explicit reward and may, in fact, receive punishment. One possibility, suggested by RL modeling, is that for many subjects the no-feedback outcome is positively valued, similar to a reward; therefore, punishment-based trials do in fact carry the possibility of a rewarding outcome. This may support continued categorization responding on these trials, reducing motivation to avoid. Another possibility is that, unlike the no-feedback outcome on classification trials, the absence of feedback on avoided trials is aversive, possibly due to uncertainty: in effect, the subject will never know what the correct answer would have been on that trial. If the latter interpretation is correct, then this raises the possibility that decreasing uncertainty on avoided trials will impact—specifically increase—avoidance behavior.

In the current study, we examine these issues further. First, we collect empirical data comparing the “no-feedback” version of the task, which allows avoidance responses but provides only an acknowledgment of the response, to a new “feedback” variant where avoidance responses are followed by informational feedback about the optimal categorization response; the rationale for the new task variant is to minimize uncertainty of outcome on avoided trials. If the uncertainty is aversive, then this manipulation should increase the rate of avoidance behavior. Second, we use the RL model methods previously employed to simulate data from the “standard” version of the task [10], and extend this model to address data from the “no-feedback” and “feedback” versions of the task, to determine whether individual differences in estimated parameters can explain the different behavioral performance when avoidance does and does not trigger informational feedback.

## Methods

### Participants

Participants were 200 young adults (Rutgers University undergraduate students, mean age 20.0 years, SD 1.8; 50.5% female). Some subjects were recruited via a departmental subject pool and received course credit in exchange for participating; other subjects were recruited via flyers around campus and were paid $20 for participating in a one-hour session. No differences on any measures were observed between subjects who participated for class credit vs. for payment. The study was approved by the Rutgers University IRB; procedures followed guidelines established by Rutgers University and the Declaration of Helsinki for the protection of human subjects. All subjects provided written informed consent before initiation of any experimental procedures.

Subjects were randomly but evenly assigned to no-feedback and feedback conditions (n = 100 per condition). One subject in the avoidance condition failed to complete all 160 trials due to experimental error; this subject’s data were excluded from analysis, leaving n = 99 in the avoidance condition.

### Procedure

**Fig 1:**
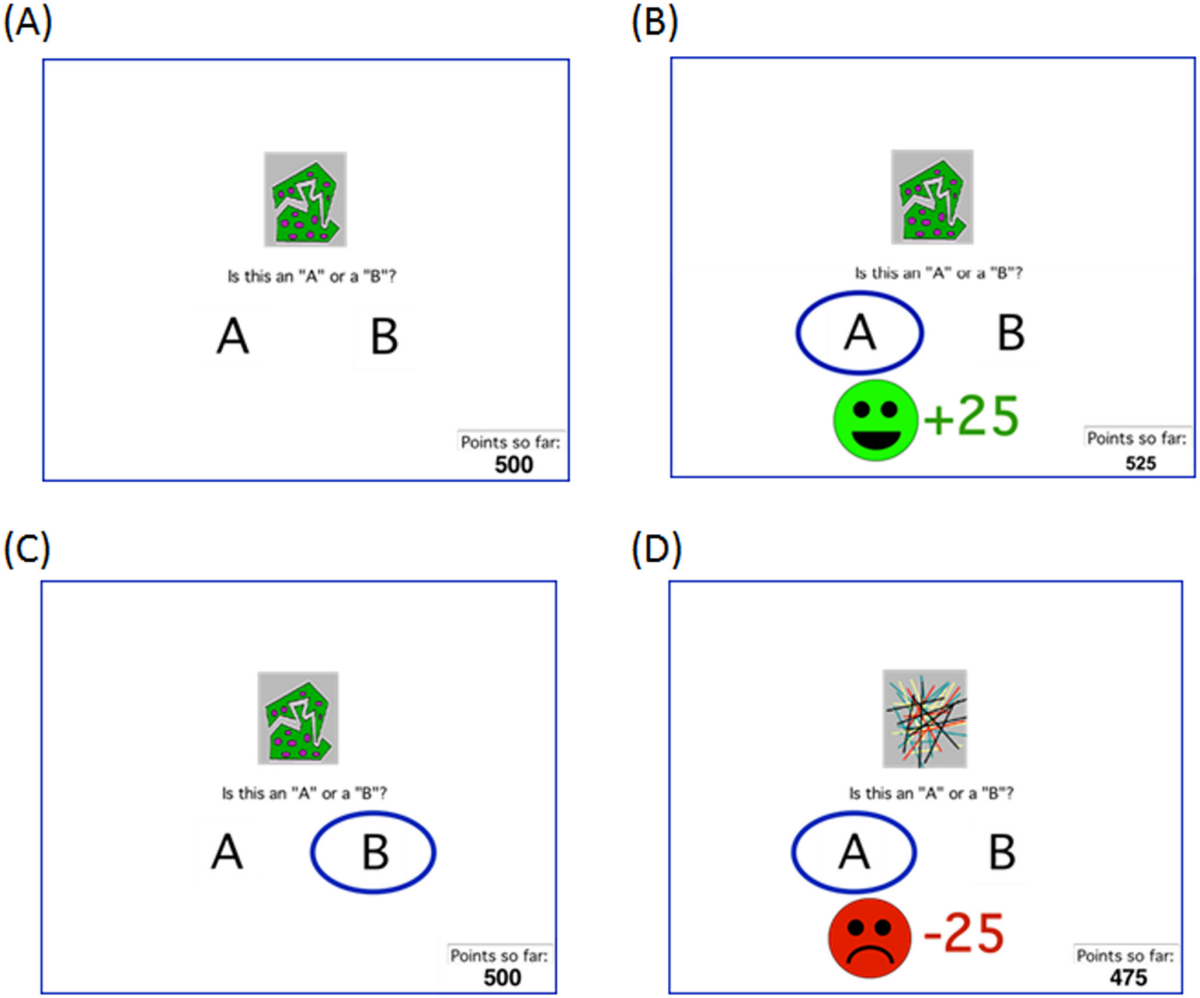
Example screen events. (A) On each trial, the participant sees a stimulus and is asked to classify the stimulus as belonging to category “A” or “B,” or to “skip”(avoid) the trial. If the subject makes a classification response, the chosen category is then circled. (B) For reward-based stimuli, correct responses are rewarded with feedback and point gain, while (C) incorrect responses receive no feedback. (D) For punishment-based stimuli, incorrect responses are punished with feedback and point loss; incorrect responses receive no feedback (similar to C). If the subject makes an avoidance response, the computer simply acknowledges the response and moves on to the next trial.

The “no-feedback” condition followed methods previously presented in Sheynin et al. [8]. Experiment 2; in brief, presentations of four stimuli were intermixed across 160 trials. On each trial, the subject saw one stimulus and was prompted to classify it as belonging to category "A" or "B" using labeled keyboard keys (Fig 1A). Two stimuli were "reward" stimuli; each was associated with one category ("A" or "B") on 80% of trials and with the opposite category on 20% of trials (Table 1). Correct categorization responses were rewarded with feedback (“You win”) and gain of 25 points (Fig 1B); incorrect categorization responses received no feedback or point gain (Fig 1C). The remaining two stimuli were "punishment" stimuli; each was associated with one category on 80% of trials and with the opposite category on 20% of trials. Incorrect responses were rewarded with feedback (“You lose”) and loss of 25 points; correct responses received no feedback or point loss. Thus, the "no-feedback" outcome was ambiguous, since it could signal missed opportunity for reward or successful avoidance of punishment. Assignment of stimuli to categories and valences was counterbalanced across trials. For each trial, subjects were scored as having made the optimal response if they chose the category that was usually correct for that stimulus, regardless of actual outcome on that trial; total points accumulated was also recorded for each subject.

**Table 1:** Category membership probabilities and outcomes for the four stimuli.

Stimulus	Category membership	Outcome/feedback
S1	80% “A”/20% “B”	Correct → +25; incorrect → no feedback
S2	20% “A”/80% “B”	Correct → +25; incorrect → no feedback
S3	80% “A”/20% “B”	Correct → no feedback; Incorrect → -25
S4	20% “A”/80% “B”	Correct → no feedback; Incorrect → -25

At the start of the experiment, subjects were informed that they also had a third option on each trial, which was simply to press a key marked “skip” to avoid the trial, in which case they would neither gain nor lose any points. Following any avoidance response, the computer simply acknowledged the response (*“OK*, *skipping this one*.*”*) and moved on to the next trial without any gain or loss of points.

The “feedback” condition was similar, except that when a skipping response was made, the computer screen circled the category that would have provided feedback on that trial; this was the optimal category on 80% of reward trials and the non-optimal category on 80% of punishment trials. For a reward trial, the feedback said, "*Skipping this one*… *If you had chosen this answer*, *you would have won 25 points*." For a punishment trial, the feedback said, "*Skipping this one*… *If you had chosen this answer*, *you would have lost 25 points*." The no-feedback outcome was therefore eliminated on avoided trials, although it could still be obtained on trials where the subject made a classification response.

Instructions for the “feedback” condition was the same as for the “no-feedback” condition, except that during practice, subjects in this group saw an example avoidance trial with the text, “*If you skip*, *sometimes the computer will show you an answer and tell you what* *would have happened* *if you had picked that answer*.”

### Data Analysis

Raw data as output from the computer-based task are provided in the supplemental material accompanying this article (S1 File). Following methods in Sheynin et al. [8], data were analyzed based on percent optimal responding to reward-based and punishment-based trials, using a mixed ANOVA with within-subjects variable of trial-type (reward vs. punishment) and between-subjects variable of condition (avoidance vs. feedback). Similar analysis was conducted on number of avoidance (“skip”) responses to reward-based and punishment-based trials. Following the Sheynin et al. study, we also calculated “adaptive responses,” defined as the percent optimal classification for reward-based trials, and the percent optimal classification, plus avoidance responses for punishment-based trials, since skipping these trials eliminates all risk of punishment. These data were analyzed using the same methods as for analyzing the optimal responses. For each of these ANOVAs, to follow up potential interactions between trial type and condition, we conducted post-hoc pairwise t-tests. Where multiple t-tests were conducted, we followed standard statistical practice of applying methods to protect against inflated risk of type I error. Specifically, we applied the Bonferroni correction which reduces alpha (the threshold for significance) by a factor equal to the number of tests conducted (e.g., for two tests, reduce alpha from .05 to .025).

We used Fisher’s exact test to determine whether the two task conditions had similar distribution of avoiders (subjects who made at least one avoidance response) and non-avoiders, or between frequent avoiders (who showed avoidance responses on at least 10% of trials) vs. infrequent avoiders and non-avoiders.

Finally, we also examined reaction time (RT) in msec for categorization responses to different trial types (reward vs. punishment) and responses (optimal response vs. non-optimal response) in each condition, via a 2 (trial type) x 2 (response type) x 2 (condition) mixed ANOVA. Again, Bonferroni-corrected pairwise t-tests were used to follow up on any significant interactions. RT for avoidance responses was also examined via 2 (trial type) x 2 (condition) mixed ANOVA; however, this analysis only included subjects who made at least one avoidance response to each trial type. Because many subjects made avoidance responses to one but not both trial types, we also conducted t-tests separately on each trial type, with factor of condition.

Because both ANOVA and t-test are parametric tests, i.e. require that the underlying data are approximately normally distributed and that groups are of approximately equal variance, we first tested the data to verify these assumptions. Where the data violated these assumptions, we used the Greenhouse-Geisser correction (for ANOVA) and Welch’s t (for t-test); each of these is a widely-used procedure which adjusts degrees of freedom to protect significance levels when assumptions are violated.

### Computational Modeling

We extended the RL-learning model from Myers et al. [10] to these data to generate estimated parameters for each participant, by incorporating a third response option (“skip”) in addition to the two categorization responses.

Full code for the model is provided in the supplemental material accompanying this article (S2 File). In brief, on each trial *t* where a stimulus *s* was presented, three expectancy values *Q[A*,*s]*, *Q[B*,*s]* and *Q[skip*,*s]* represented the expected outcomes if stimulus *s* were responded to with category A or B, or skipped (avoided). All *Q* were initialized to 0 at the start of a simulation run. On each trial *t* = 1..160, the probability *Pr(r*,*t)* of response *r* (A, B, or skip) was calculated using a softmax function:

$$\text{Pr}\left(\text{r},\text{t}\right)=\frac{{\text{e}}^{\text{Q}\left[\text{r},\text{s}\right]/\text{T}}}{{\text{e}}^{\text{Q}\left[\text{A},\text{s}\right]/\text{T}}+{\text{e}}^{\text{Q}\left[\text{B},\text{s}\right]/\text{T}}+{\text{e}}^{\text{Q}\left[\text{skip},\text{s}\right]/\text{T}}}$$ (1)

where *T* was a “temperature” parameter that could range from 0..1 and that specified the tendency to choose the response with highest expectancy value (low *T*) or choose a response at random (high *T*). Note that *Pr(A*,*t)+Pr(B*,*t)+Pr(skip*,*t)* = 1.

Prediction error *PE* was computed as *PE = R-Q[r*,*s]* where *r* was the participant’s actual response on that trial (category A or B), *s* was the current stimulus, and *R* was the feedback that the participant received on that trial. As in prior modeling [10], following a classification response, *R* could take one of three values: *R+* (reward), *R-* (punishment), or *R0* (no feedback). *R+* was fixed at +1 and *R-* at -1. Prior work with this task, including the veteran study described above [10], indicated important individual and group differences in how subjects valued the no-feedback outcome, with some subjects valuing it as strongly positive (similar to reward, i.e. approaching +1), and others valuing it as strongly negative (similar to punishment, i.e. approaching -1). As a result, here also we considered *R0* as a free parameter that could vary from -1 (same as explicit punishment) to +1 (same as explicit reward).

Finally, the feedback version of the task provided different outcomes to reward-based and punishment-based trials (i.e., “you would have won…” vs. “you would have lost…”). It was conceivable that subjects would value these in opposite directions. Thus, for example, information that “you would have lost” could be viewed as describing a missed punishment (hence, positively valued and increasing the likelihood of repeating the avoidance response in future) or as conveying information about the correct categorization response (hence, increasing the likelihood of categorizing this stimulus in the future, and thereby decreasing the likelihood of avoiding). The informational feedback could also be considered as purely informational, and not affecting operant responses in the task (i.e., neither positively nor negatively valued). To capture these possibilities, we also considered two additional free parameters, *RSrew* and *RSpun*, which were the value of feedback following a skip response on reward-based or punishment-based trials, respectively, and which could also vary independently from -1 to +1.

Expectancy values associated with the current values of *r* and *s* were then updated based on whether the outcome *R* was better (*PE*>0) or worse (*PE*<0) than expected:

$$\begin{array}{ll} \text{Q}\left[\text{r},\text{s}\right]\leftarrow \text{Q}\left[\text{r},\text{s}\right]+\text{ PE}*\text{LR}+ & \text{if PE}>0\\ \text{Q}\left[\text{r},\text{s}\right]\leftarrow \text{Q}\left[\text{r},\text{s}\right]+\text{ PE}*\text{LR}- & \text{if PE}<0\\ \text{Q}\left[\text{r},\text{s}]\;\leftarrow \text{Q}[\text{r},\text{s}\right] & \text{if PE}=0 \end{array}$$ (2)

where *LR+* and *LR-* were the learning rates associated with gain and loss trials respectively [18], and could each range from 0 to 1.

Following the model used in Myers et al. [10], four free parameters were explored: the learning rate *LR+* for trials where the outcome was better than expected; the learning rate *LR-* for trials where the outcome was worse than expected; the explore/exploit parameter *T* which specified the tendency to repeat previously-successful responses vs. try a different response, and the reinforcement value *R0* of the no-feedback outcome. For each subject, the model was run with each value of *LR+*, *LR-*, and *T*, ranging from 0..1 in step sizes of 0.05, and *R0* from -1 (*R-*) to +1 (*R+*) in step sizes of 0.1.

In the “feedback” condition, skipping responses triggered informational feedback, which was different on reward-based and punishment-based trials (*RSrew* and *RSpun*, respectively), and thus might be differentially valued. Specifically, avoidance in punishment-based trials triggers feedback about potential loss, which might be positively valued (signaling successful avoidance of that potential loss), while avoidance on reward-based trials triggers feedback about potential reward, which might be negatively-valued (signaling failure to obtain that potential reward). Accordingly, we also considered a six-parameter model where the value of *RS* could differ for reward and punishment-based trials. In this case, two free parameters *RSrew* and *RSpun* were independently allowed to vary from -1…+1 in increments of 0.1. This model therefore contained six free parameters (*LR+*, *LR-*, *T*, *R0*, *RSrew*, *RSpun*). We applied this model to data from both experimental conditions, even though we had no *a priori* reason to believe that estimated values *RSrew* and *RSpun* would differ in the avoidance condition, where avoidance response triggered the same acknowledgment (*“OK… skipping this one…*”) regardless of trial type.

By comparison to this “full” six-parameter model, we refer to the earlier model from Myers et al. [10] as the “no *RS*” model, since it is equivalent to the full model with *RSrew* and *RSpun* fixed to 0. Note that these two models are identical for subjects who never made an avoidance response, and therefore never experienced this type of feedback, and the best-fit value of *RSrew* and *RSpun* are undefined for these subjects.

In addition, we also investigated several simpler versions where other parameters were held constant: a five-parameter model with a single learning rate (*LR+* = *LR-*), a four-parameter model with a single value of *RS* (*RSrew = RSpun*), a three-parameter model with *R0 = RSrew = RSpu*n = 0, and a five parameter model with *T* held constant at 0.23, which was the mean value observed for *T* under the six-parameter model. Finally, based on simulations that suggested both the earlier (no *RS*) model and the model with fixed *T* performed well, we considered a model that held both RS and T fixed, leaving only *LR+*, *LR-* and *R0* as free parameters. Table 2 summarizes the models examined. (Other simpler models were also explored but did not perform better than these models, and are not discussed further here.)

**Table 2:** Description and free parameters in each of the six models explored.

Model Name	Description	Free Parameters
“Full” model	Full model with six free parameters	6 (*LR+*, *LR-*, *T*, *R0*, *RSrew*, *RSpun*)
No *RS*	*RSrew = RSpun* = 0 (same as ref [10])	4 (*LR+*, *LR-*, *T*, *R0*)
One *RS*	*RSrew = RSpun*	5 (*LR+*, *LR-*, *T*, *R0*, *RS*)
One *LR*	Single learning rate: *LR = LR+ = LR-*	5 (*LR*, *T*, *R0*, *RSrew*, *RSpun*)
Fixed *T*	*T* set at mean value of 0.23	5 (*LR+*, *LR-*, *R0*, *RSrew*, *RSpun*)
No *R0*	*R0* = 0 (and *RSrew = RSpun* = 0 also)	3 (*LR+*, *LR-*, *T*)

For each subject under each model, model fit was assessed by computing negative log likelihood estimates (LLE) to estimate the a priori probability of the data, given a particular combination of parameter values:

$$negLLE=-\sum_{t=1..160}logPr\left(r,t\right)$$ (3)

where *r* is the response made by the subject on trial *t* (i.e., *Pr(r*,*t)* is the probability that the model makes the same response as the subject on that trial). Estimated parameters for each participant were defined as the values of *LR+*, *LR-*, *T*, *R0*, *RSrew* and *RSpun* that together resulted in the lowest *negLLE* for that participant’s data. Note that, with three response options, a truly random model would generate *negLLE* = -160*ln(0.33) = 175.8, while a model that perfectly described the subject’s trial-by-trial responses would generate *negLLE* = 0.

To compare models, we used the Bayesian Information Criterion (BIC; [20]), which penalizes models with more free parameters: BIC = k*ln(n)+2*negLLE, where *k* is the number of free parameters and *n* is the number of observations (here, *n* = 160); lower values of *BIC* indicate better fit. We then used the random effects Bayesian model selection procedure [21, 22] which takes into account the possibility that different models may have generated different subjects’ data, to generate expected posterior probabilities for each model.

Models were compared using Bayesian model selection (BMS), using the following toolbox: http://mbb-team.github.io/VBA-toolbox/. A variational Bayes method originally described by Stephan et al. (2009) was implemented using VBA (Daunizeau et al. 2014). This method estimates the frequency that any model was most likely to have generated the data from any subject, as well as the exceedance probability, which is the probability of each model having the highest frequency. Given the conditional probabilities of the models (*ri*), the exceedance probability of any one model can be calculated as:

$$\varphi_{i}=p\text{(}r_{i}>\;r_{j}|y;\alpha )$$ (4)

Where *r*_*j*_ is the conditional probability of any model not equal to *r*_*i*_ given the observed data *y* and the Dirichlet parameters,.The exceedance probabilities for all models tested therefore sum to 1.

Finally, given a “winning” model, we recorded estimated parameter values for each subject under that model, and compared values across experimental condition (no-feedback vs. feedback), as well as based on whether the subject avoided at least one trial (“avoider”) or never avoided any trials (“non-avoider”). Because the data were highly skewed (due to boundaries on parameter space explored), non-parametric tests were used (e.g. Mann-Whitney U as a non-parametric alternative to independent-samples t-test, Wilcoxon Signed-Rank test as a non-parametric alternative to paired-samples t-test, and Kendall’s τ_b_ as a non-parametric alternative to Pearson’s correlation where number of tied scores is high). Where multiple tests were conducted, Bonferroni correction was applied to reduce alpha to protect against inflated risk of Type I error.

## Results

### Empirical Data

**Fig 2:**
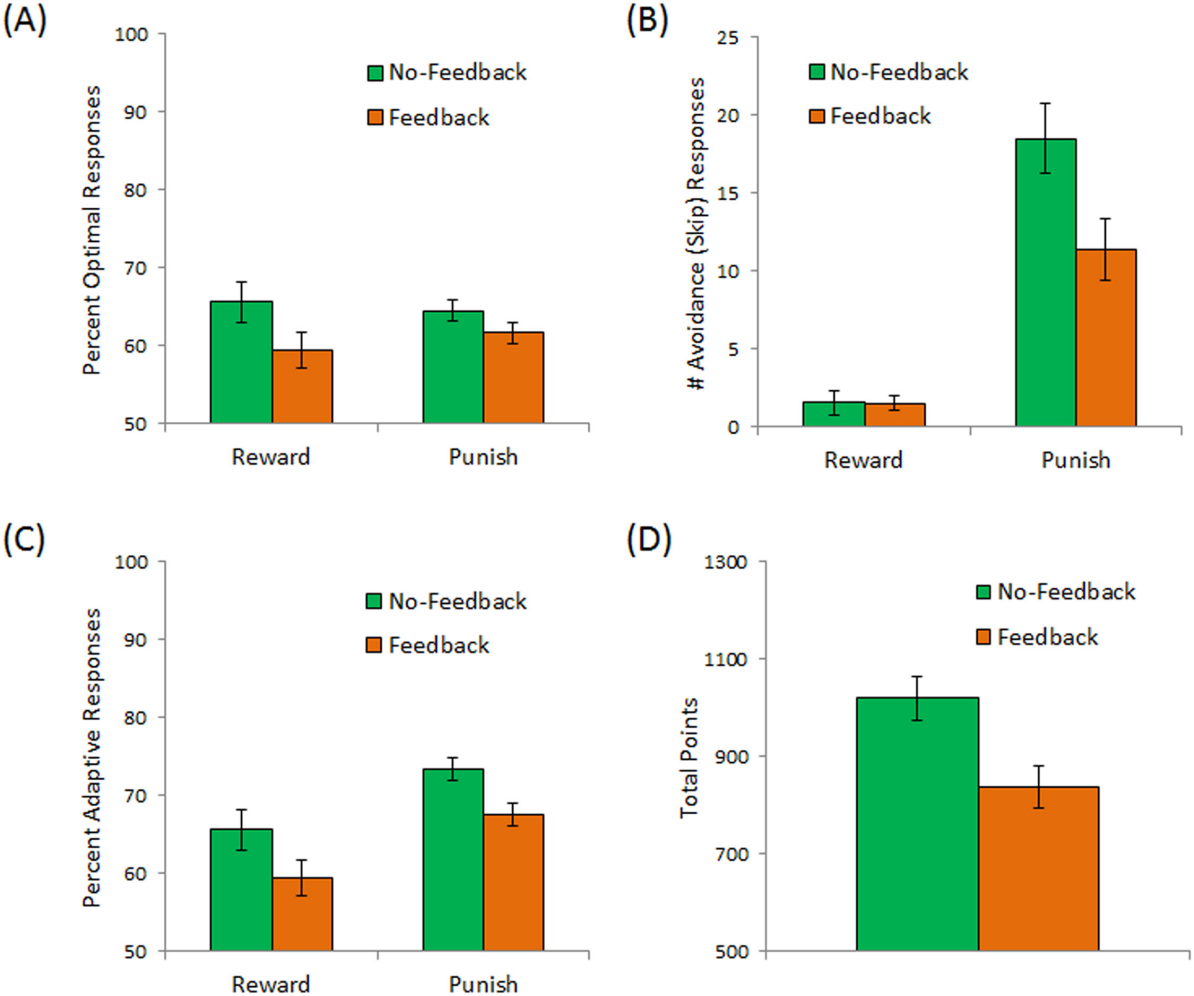
Empirical data from the no-feedback condition (green bars), in which subjects could avoid categorization by “skipping” the trial, and the feedback condition (orange bars) in which avoidance responses generated informational feedback. Subjects in the no-feedback condition made more optimal classification responses (A) and avoided (skipped) more punishment-based trials (B) than subjects in the feedback condition. When these measures were combined into an overall measure of adaptive responses (optimal classification plus avoidance of punishment-based trials), subjects the no-feedback condition showed better performance on both reward-based and punishment-based trials (C), resulting in significantly more total points accrued by subjects in the no-feedback condition (D). In this and subsequent figures, error bars represent SEM.

Repeated-measures ANOVA on performance, scored as percent optimal responses to reward and punishment stimuli, revealed a significant effect of condition (Fig 2A), with subjects in the no-feedback condition significantly outperforming those in the feedback condition (F(1,197) = 4.69, p = .032) but no difference in performance on reward vs. punishment trials (F(1,197) = 0.10, p = .752) and no condition x trial-type interaction (F(1,197) = 0.90, p = .344). As shown in Fig 2B, the overwhelming majority of avoidance responses were to punishment-based stimuli (F(1,197) = 88.14, p < .001); there was also a main effect of condition (F(1,197) = 4.35, p = .038) and a condition x trial-type interaction (F(1,197) = 6.17, p = .014). Post-hoc t-tests, with alpha adjusted to .025 to protect significance levels, revealed significantly more avoidance responses on punishment-based trials in the no-feedback condition than in the feedback condition (Welch’s t(193.10) = 2.37, p = .019), but no difference between conditions on reward-based trials (t(197) = 0.02, p = 0.988).

As in Sheynin et al. [8], many subjects did not make any avoidance responses during the experiment. Specifically, 38.0% of subjects in the no-feedback condition, and 50.5% of subjects in the feedback condition made no avoidance responses at all; this was not a significant difference between conditions (Fisher’s exact test, two-tailed, p = .087).

Turning to percent adaptive responses, there was again a main effect of condition, with subjects in the no-feedback condition outperforming those in the feedback condition (Fig 2C; F(1,197) = 7.37, p = .007); there was also an effect of trial type (F(1,197) = 21.44, p < .001), with subjects performing better on punishment-based than reward-based trials, but no condition x trial type interaction (F(1,197) = 0.02, p = .897). This effect of condition resulted in subjects in the no-feedback condition accruing significantly more total points than those in the feedback condition (Fig 2D; t(197) = 2.89, p = .004).

**Fig 3:**
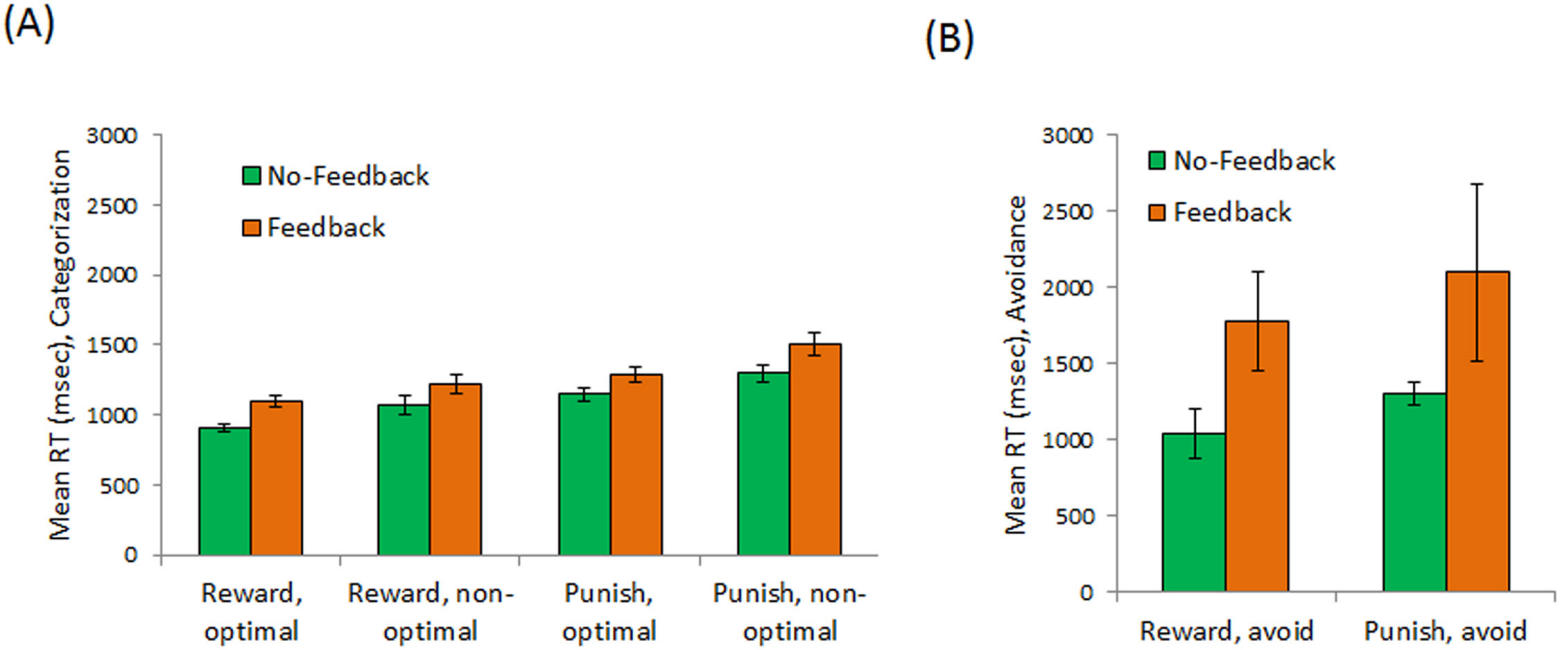
Reaction time (RT) measures. (A) On trials where a classification response was made, there was generally slower responding on punishment than reward trials and on trials where the non-optimal response was made; there was also a main effect of task condition, with subjects in the no-feedback condition generally responding faster than subjects in the feedback condition. (B) On trials where an avoidance response was made, there were no significant differences between reward-based and punishment-based trials, but subjects in the no-feedback condition again responded faster overall than subjects in the feedback condition.

Finally, Fig 3A shows mean RT on trials where a classification response was made. There were expected effects of trial type, with slower responding on punishment-based than reward-based trials (F(1,182) = 62.03, p < .001), and response type, with slower responding when the response was non-optimal than when it was optimal (F(1,182) = 36.35, p < .001). There was also a main effect of condition, with subjects in the no-feedback condition responding faster overall than subjects in the feedback condition (F(1,182) = 8.02, p = .005). There were no significant interactions among any of these variables (all p>.200).

Fig 3B shows mean RT on trials where an avoidance response was made. Only 36 subjects (19 in each condition) avoided at least one reward-based and at least one punishment-based trial; among these subjects, RT on avoided trials showed a significant interaction between trial type and condition (F(1,36) = 5.47, p = .025), with no main effect of trial type (F(1,36) = 1.75, p = .194) or condition (F(1,36) = 3.07, p = .088). Considering just subjects who made at least one avoidance response on a reward-based trial (21 in each condition), there was significantly faster avoidance responding by subjects in the no-feedback than feedback condition (t(40) = 2.05, p = .047). Considering just subjects who made at least one avoidance response on a punishment-based trial (60 subjects in the no-feedback condition and 47 in the feedback condition), there was no significant difference in RT on avoidance responses (t(105) = 1.54, p = .127). The difference is still not significant after excluding one response by a subject in the feedback condition which took 27 sec (the next longest RT was about 7.5 sec).

Thus, empirical data from the no-feedback condition generally reproduce the behavioral data from the Sheynin et al. study, suggesting that the conclusions hold across samples, at least from this college population. However, the results in Fig 2 indicate that provision of informational feedback on avoided trials reduces the tendency to avoid (particularly punishment trials) and results in overall poorer performance on the task. We turn next to the RL model, to better understand why this is so.

### Modeling Results

**Fig 4:**
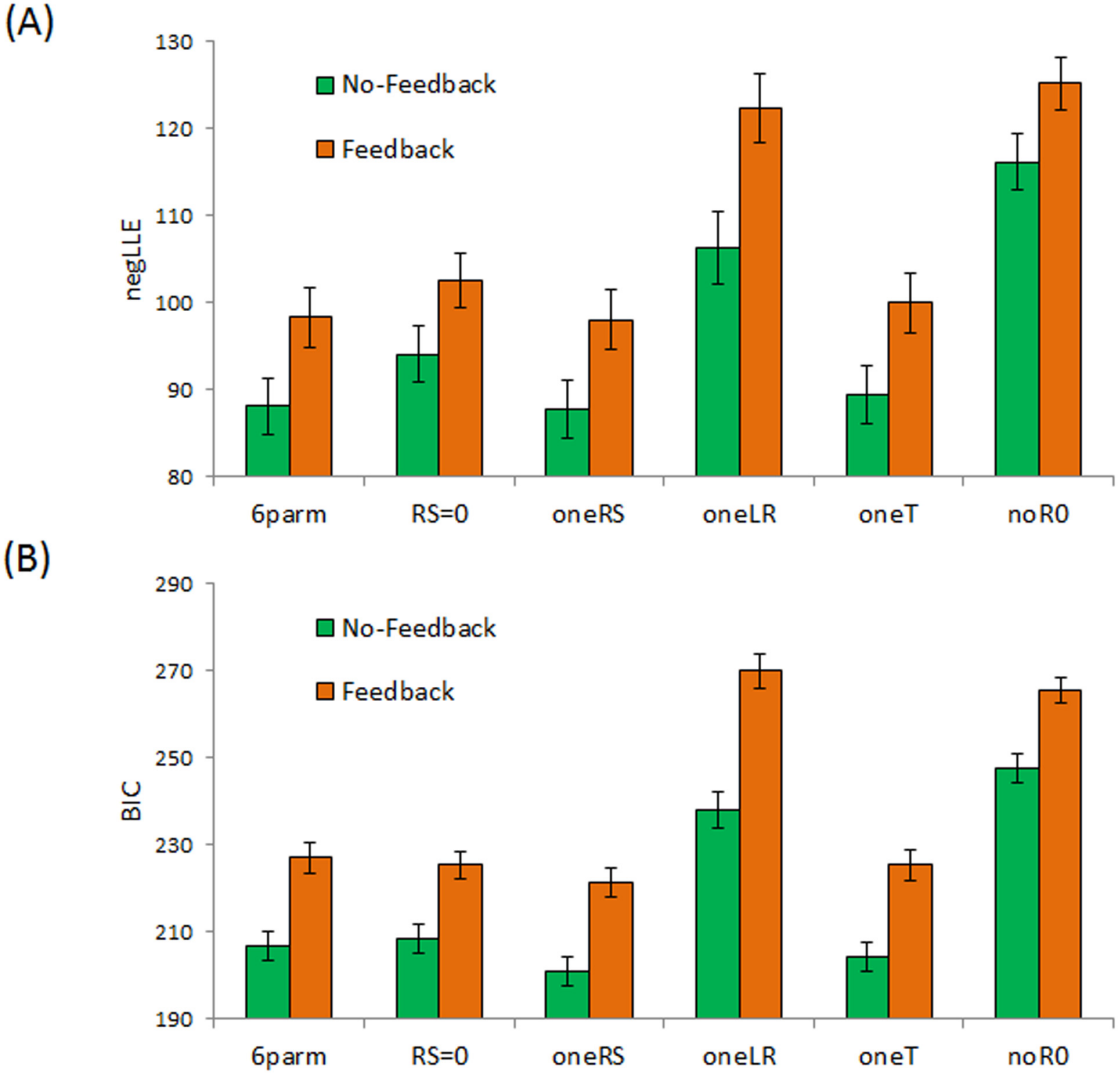
Model fit in terms of (A) *negLLE* and (B) *BIC* for the six models from Table 1, applied to data from subjects in the no-feedback and feedback conditions.

Fig 4A shows that, on average, *negLLE* is numerically lower for all models applied to data from the no-feedback than the feedback condition. There are also differences across models. To formally compare models, we computed *BIC* which rewards model fit while penalizing model complexity. Fig 4B shows that, while the full model with 6 free parameters fared well (low *BIC*) on average, the four-parameter model from our prior study (*RS* = 0) also fared well, as did the models with a single *RS* or a fixed value of *T*; by contrast, models with only a single *LR* or with *R0* fixed at 0 fared worse.

In addition, although Fig 4A shows average values of *negLLE*, there was considerable individual variability. First, note that for the *n* = 88 non-avoiders, *negLLE* for the full 6-parameter model, the *RS = 0* model, and the *oneRS* model was numerically identical: since these subjects never made a single skip response, they never experienced either *RSrew* or *RSpun* and so for them the value of these parameters was moot. As a result, for all non-avoiders, lowest *BIC* was obtained under the *RS = 0* model, which had fewer free parameters (*k* = 4) compared to the *oneRS* (*k* = 5) or full 6-parameter model (*k* = 6).

The situation was very similar for the *n* = 36 subjects who made only a few (<10) avoidance responses in the experiment, for whom these three models again generated very similar *negLLE*. In these cases, *BIC* was again slightly higher for the full 6-parameter model, reflecting the fact that *BIC* modestly penalizes more complex models. Thus, for 32 of these 36 “infrequently-avoiding” subjects, the *RS = 0* model produced lowest *BIC* among all the models.

However, among the remaining 75 subjects who made frequent (≥10) avoidance responses, the RS = 0 model fared poorly, both in terms of negLLE and BIC, producing the lowest BIC for only 4 of the 75 subjects.

As a result of these differences across models, Bayesian analysis comparing all the models yielded exceedance probabilities of 0.9999 for the six-parameter model, and 0.0001 for all other models. Based on these results, we consider the six-parameter model further below.

For most subjects, unique configurations of parameter values were derived that provided “best-fit” to that subject’s data, in terms of minimizing *negLLE*. However, in some cases “best-fit” was obtained with a range of values for one or more of the parameters; this included *R0* (n = 7 subjects equally fit for a given range of parameter values), *RSrew* (n = 19), and *RSpun* (n = 19). This occurred for 19 subjects in the no-feedback condition and 20 subjects in the feedback condition (numbers do not sum to 45 due to some subjects generating a range of “best-fit” values for more than one parameter). In these cases, the estimated value for the parameter was assigned as the median of that range (range often 0..-1 but never included any value >0, so that median always <0).

**Fig 5:**
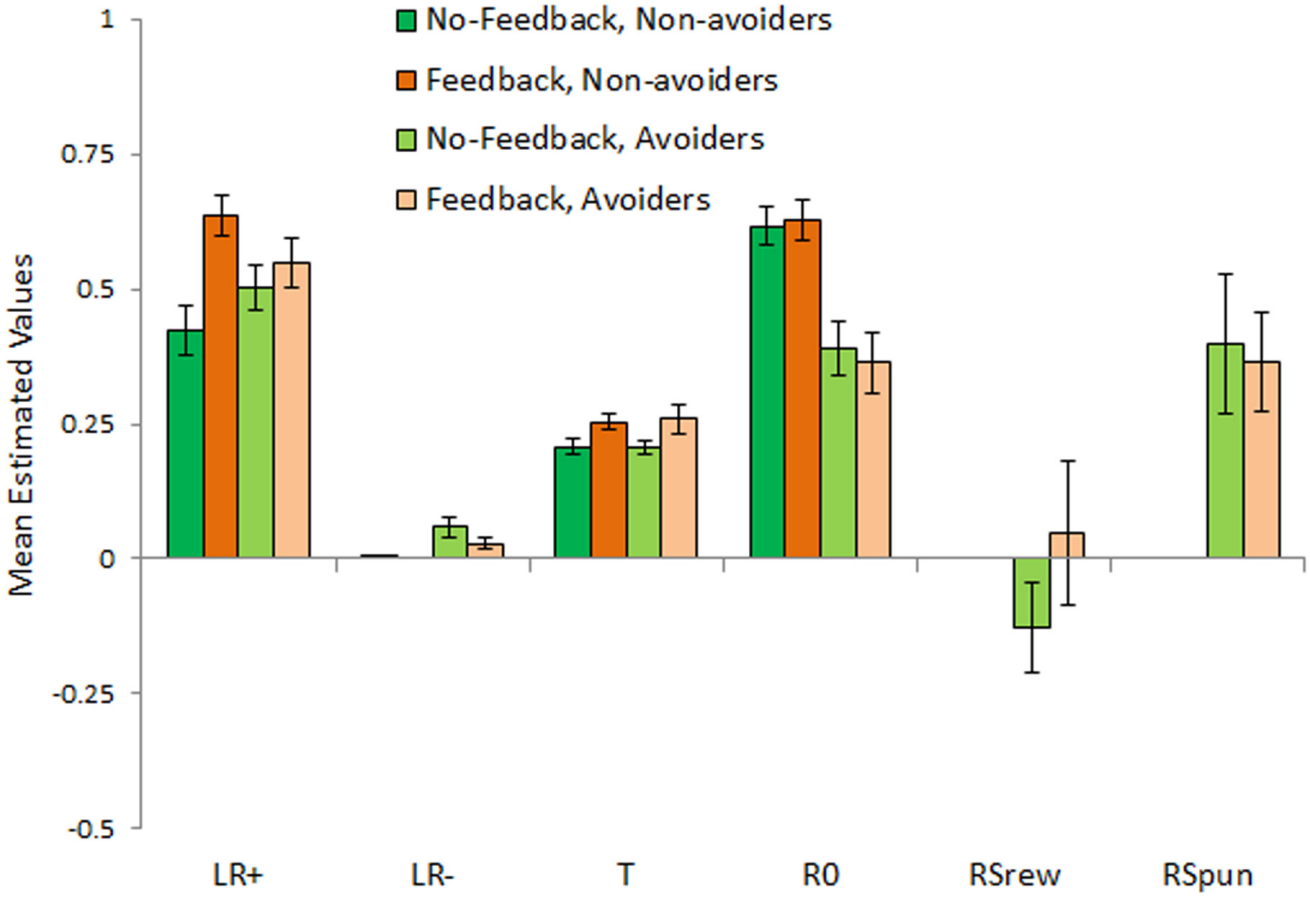
Results obtained with the six-parameter model. Overall, subjects in the no-feedback condition had larger estimated values of *LR+*, indicating that they adjusted their behavior faster following better-than-expected feedback, compared to subjects in the feedback condition. In addition, estimated values of *R0* were significantly lower, and those of *LR-* were significantly higher, in subjects who made at least one avoidance response (“avoiders”), compared to subjects who made no avoidance responses at all (“non-avoiders”), regardless of experimental condition. (Note that estimated values for *RSrew* and *RSpun* are undefined for non-avoiders, who never experienced these types of feedback.)

Fig 5 shows mean estimated parameter values; since the data were non-normal, non-parametric Mann-Whitney tests were used to compare conditions, with alpha adjusted to .05/6 = .0083 to protect significance. Estimated values of *LR+* were significantly greater in the feedback than the no-feedback condition (Mann-Whitney U = 3837.5, p = .006). This indicates that subjects in the feedback condition adjusted their behavior faster following better-than-expected feedback, compared to subjects in the no-feedback condition. There was no significant difference between conditions in estimated values of *LR-* (U = 4518.0, p = .087), *T* (U = 4113.5, p = .030), *R0* (U = 4706.0, p = .546), *RSrew* (U = 177.0, p = .257), or *RSpun* (U = 1358.5, p = .742).

Fig 5 also shows that estimated values of *R0* were significantly greater in subjects who made no avoidance responses than in subjects who made at least one avoidance response (U = 2894.5, p < .001) while values of *LR*- were significantly lower in avoiders than non-avoiders (U = 3658.0, p < .001). There were no differences between avoiders and non-avoiders in estimated values of *LR*+ (U = 4669.5, p = .593) or *T* (U = 4438.5, p = .245). (*RSrew* and *RSpun* are undefined for non-avoiders, who never experienced these types of feedback.) The significant differences in *R0* and *LR-* indicate that non-avoiders adjusted their behavior slower following worse-than-expected feedback, and also valued ambiguous feedback more positively, compared to avoiders.

Finally, as suggested by Fig 5, estimated parameter values within subjects were generally larger for *LR+* than *LR-* (Wilcoxon paired-samples test, W = 146.5, Z = -11.858, p < .001) and for *RSpun* than *RSrew* (W = 98, Z = -3.104, p = .002).

**Fig 6:**
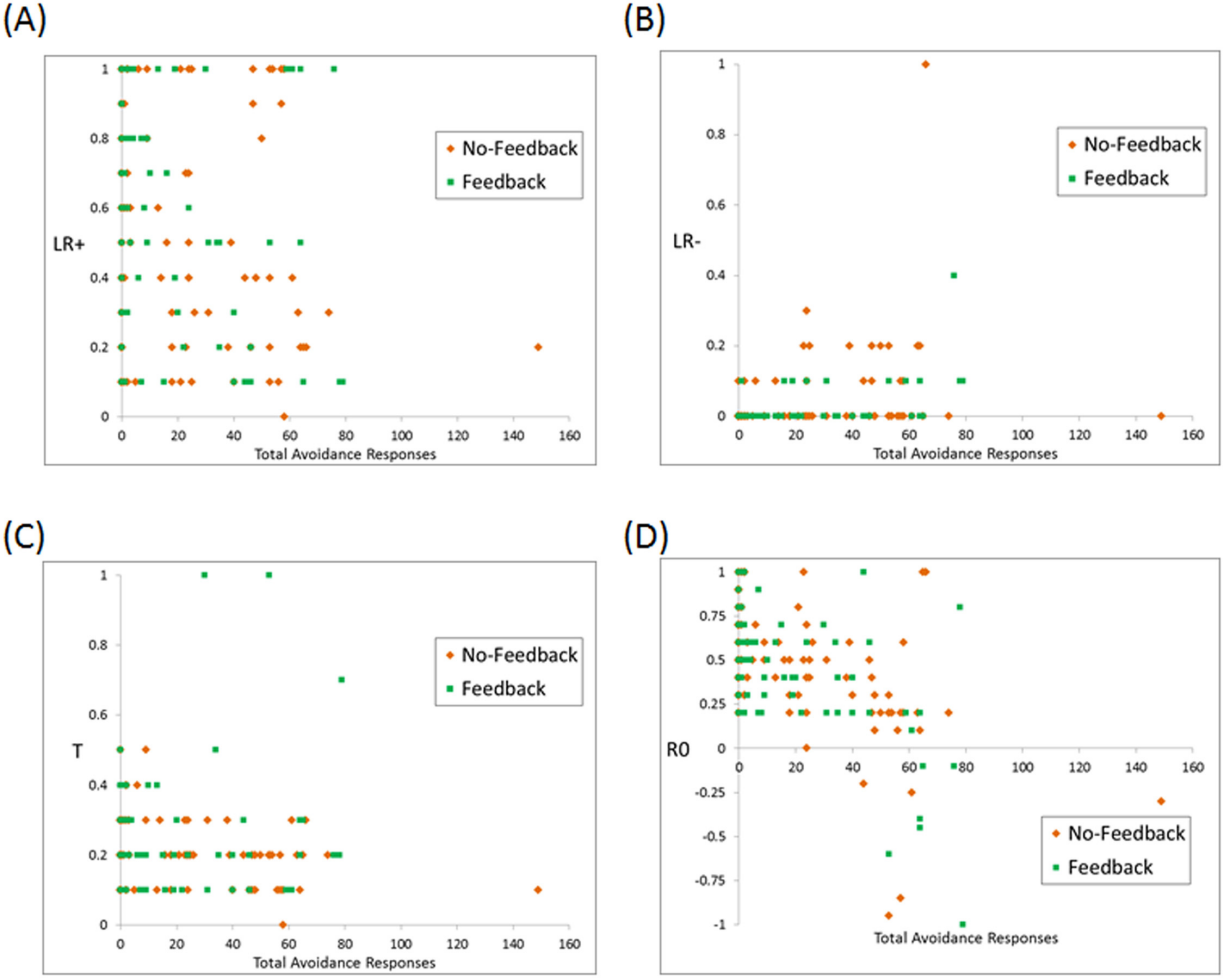
Correlations between avoidance behavior and estimated parameter values (A) *LR+*, (B) *LR-*, (C) *T*, (D) *R0*. *LR*- was positively correlated with avoidance responses, indicating that subjects who made more avoidance responses tended to learn more quickly from worse-than-expected feedback; *R0* was negatively correlated with avoidance responses, indicating that subjects who made more avoidance responses tended to more negatively value the ambiguous neutral feedback on categorization trials.

Dichotomizing subjects as “avoiders” vs. “non-avoiders” of course masks considerable variability, since it groups together subjects who made even a single avoidance response, those who made a few avoidance responses, and those who avoided a majority of trials. To further examine the relationship between estimated parameters and skipping behavior, Fig 6 shows estimated parameters for each subject as a function of total avoidance responses. As shown in Fig 6B, *LR*- was positively correlated with avoidance responses (τ_b_ = +.383, p < .001); i.e., subjects who made many avoidance responses tended to have faster learning following worse-than-expected feedback. Next, as shown in Fig 6C, *R0* was negatively correlated with avoidance responses (τ_b_ = -.384, p < .001); i.e., subjects who made many avoidance responses tended to more negatively value the ambiguous neutral feedback on categorization trials. Neither *LR+* nor *T* were significantly correlated with avoidance responses (*LR+*: τ_b_ = -.077, p = .148; *T*: τ_b_ = -.110, p = .054). As also shown in Fig 6, the data included one subject who made avoidance responses on 149 out of 160 trials (no other subject made more than 79 avoidance responses); when this subject’s data were excluded from analysis, the significant correlations were slightly strengthened, and the non-significant correlations slightly weakened, but otherwise results were essentially the same.

**Fig 7:**
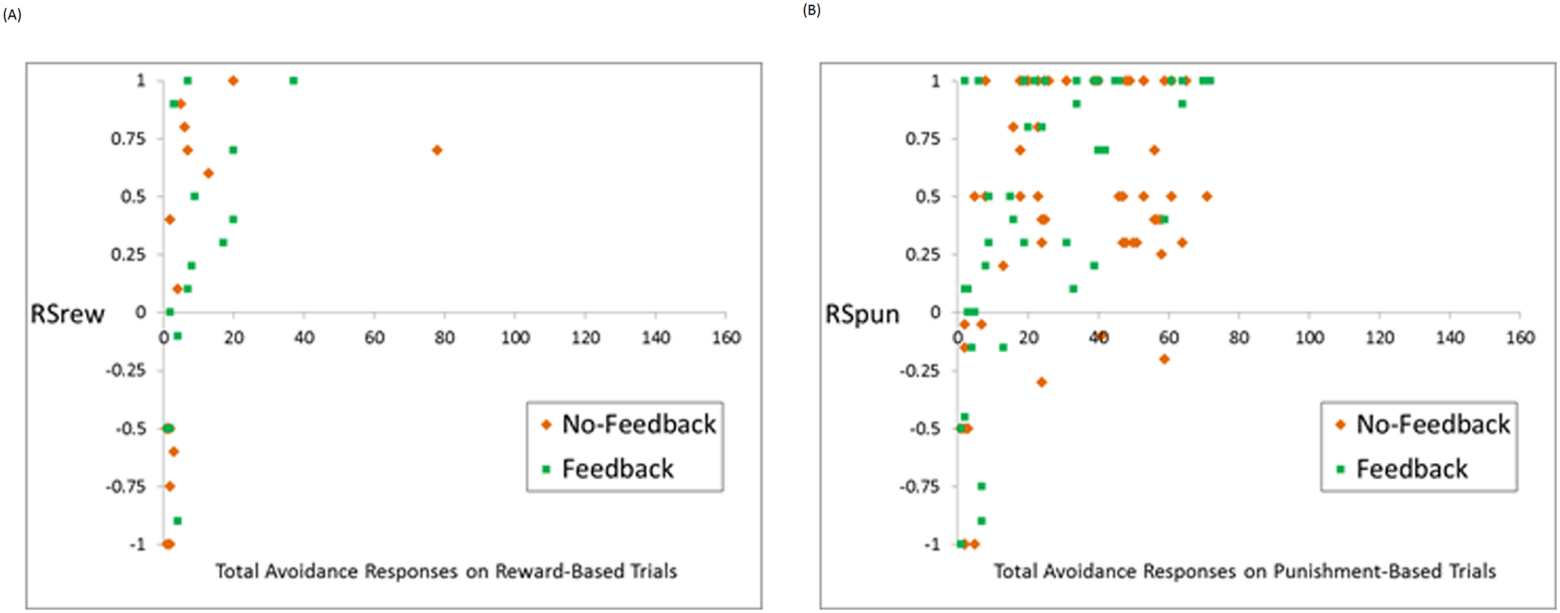
Correlations between avoidance behavior and estimated parameter values (A) *RSrew* and (B) *RSpun*, for subjects who made at least one avoidance response to the corresponding trial type, and whom these parameters therefore had defined values. Both *RSrew* and *RSpun* were positively correlated with avoidance responses on the corresponding trial types, indicating that subjects who made more avoidance responses tended to more positively value the resulting feedback.

Finally, among those subjects who made avoidance responses (and for whom *RSrew* and *RSpun* could be meaningfully defined), *RSrew* was positively correlated with number of reward-based trials avoided (Fig 7A; τ_b_ = +.554, p < .001) and *RSpun* was positively correlated with number of punishment-based trials avoided (Fig 7B; τ_b_ = +.427, p < .001); i.e., subjects who tended to skip each type of trial correlated also tended to more positively value the resulting feedback.

## Discussion

We previously showed that a simple RL model could adequately capture subject behavior from a probabilistic reward and punishment learning task [10]; later, we considered a version where, in addition to the option to classify the stimulus as "A" or "B.", subjects had the option to "skip" or avoid a trial and receive no feedback [8]. While some subjects (“avoiders”) took advantage of this option particularly on punishment-based trials, other subjects (“non-avoiders”) never made any skipping responses at all—even though avoiding is clearly the optimal response on punishment-based trials where there is a possibility of explicit punishment with no possibility of explicit reward. One possible explanation for this pattern is suggested by prior RL modeling, which suggests large individual and group differences in *R0*, the subjective valuation of the no-feedback outcome; the modeling suggested that many subjects value *R0* as strongly positive, similar to a reward; such a valuation means that rewarding feedback is, in fact, possible following successful categorization on a punishment-based trial, which in turn reduces the motivation to avoid those trials. Another possible explanation is that the lack of feedback following an avoidance response is aversive, perhaps due to subjects’ intolerance of uncertainty or curiosity about the actual answer; in either case, aversion to lack of feedback would reduce the motivation to avoid.

To further investigate this issue, we here compared a version of the task where avoidance responses generate no feedback with one where feedback is provided, specifying what the correct response would have been on reward-based trials, or what the incorrect response would have been on punishment-based trials. This manipulation reduces uncertainty following an avoidance response, and if intolerance of uncertainty is indeed biasing subjects against avoidance, this manipulation should therefore increase avoidance responding.

In fact, we found no evidence of increased avoidance responding with this manipulation. Rather, avoidance rates actually decreased, as did overall task performance assessed both as optimal behavior and as total points gained. However, consistent with our prior work, we again found large individual differences in *R0*, with subjects who valued *R0* most positively tending to make few or no avoidance responses. We address these points in more detail below.

### The role of uncertainty in avoidance

The idea that informational feedback should increase avoidance was based on the idea that many subjects are intolerant of uncertainty, and that reduction of uncertainty is inherently rewarding.

If subjects are driven by intolerance of uncertainty then, in the no-feedback condition, categorization—which can result in feedback—should be pursued at the expense of avoidance—which never results in feedback—even though categorization also carries the risk of punishment. If so, then avoidance should be considerably higher in the feedback condition.

In fact, we found exactly the opposite: results from the no-feedback condition were similar to those previously observed by Sheynin et al. [8], with a sizeable percentage of subjects never availing themselves of the avoidance response, but provision of informational feedback in the feedback condition actually reduced avoidance, without improving categorization.

Rather than supporting the idea that subjects are intolerant of uncertainty, the finding that many subjects were “non-avoiders” may be more consistent with theories suggesting that exploration and uncertainty are in fact reinforcing. For example, optimal arousal theory [23], argues that people like a certain level of arousal and uncertainty. In the no-feedback condition, this could translate into a bias to attempt categorization, even at the risk of losing points, rather than to avoid, and lose any chance of feedback. This would also potentially explain why avoidance was decreased in the feedback group, when uncertainty following an avoidance response was further reduced. Indeed, it has been previously shown that individuals often opt out of decision-making to avoid the associated information [24]; thus, when informational feedback is provided regardless of the avoidance behavior, the individual might become less motivated to avoid. It is also possible that such feedback diminished subjects’ sense of threat associated with the task, which in turn resulted in reduced avoidance [25].

Another possibility is that the provision of informational feedback could make subjects feel more confident of the correct category membership, increasing their bias to attempt categorization in the feedback condition. Arguing against this idea is the fact that, on trials where a classification response was made, subjects in the feedback version were no more accurate than subjects for whom skipping did not produce feedback. In fact, performance in the feedback version of the task was worse (reflected in fewer total points) and slower (reflected in higher RTs), despite the fact that this group had additional information to guide responding.

These ideas could be explored further in future work, by explicitly querying subjects’ confidence in their categorization responses, and by measuring subject arousal to see whether this differed across conditions and also if there were trial-by-trial changes as a function of trial outcome.

### The role of *R0*

Consistent with prior work [10], we found large individual differences in estimated values of *R0*, indicating that some subjects viewed the ambiguous no-feedback outcome as positive, similar to a successfully missed punishment (*R0* → +1), others viewed it as neutral (*R0*≈0), and still others viewed it as negative, similar to a missed reward (*R0* → -1). Model fit was considerably worse when *R0* was held as a fixed parameter, indicating that the valuation of ambiguous feedback is an important component affecting behavior.

Similarly, model fit was poor when it was assumed that there was only a single learning rate, rather than allowing different learning rates when the subject receives better-than-expected (*LR*+) or worse-than-expected (*LR*-) outcomes.

In fact, for subjects who made no avoidance responses (non-avoiders) or few avoidance responses (infrequent avoiders), the *RS = 0* model, which allowed *LR+*, *LR-*, *R0*, and *T* to vary, provided the most parsimonious description of the data, in terms of lowest *BIC*. This is similar to the conclusion from prior work [10], indicating some consistency across samples.

However, when all subjects—including those who frequently made avoidance responses—are considered, random effects model comparison suggested that the best fit—closest description of the subject data, with fewest free parameters—was obtained using a six-parameter model where individual differences could be captured by free parameters including *LR+*, *LR-*, *R0*, and *T*, as well as values of *RSrew* and *RSpun* that could vary independently.

Using this full six-parameter model, estimated parameter values for *LR*+ were significantly greater for subjects in the feedback than in the no-feedback condition (Fig 5), indicating that the former group learned faster from positive outcomes. This did not translate into better behavioral performance on either the reward-based or punishment-based trials. On the other hand, there were no differences on the other parameters between subjects in the two conditions.

Rather, regardless of task condition, *LR*- was higher and *R0* was lower in subjects who made at least one avoidance response, compared to non-avoiders. Higher *LR*- could drive subjects to avoid, rather than attempt categorization, on trials where punishment is possible; similarly, although non-avoiders typically valued *R0* as strongly positive (similar to reward), avoiders valued *R0* more neutrally. This means that, while a successful categorization response on a punishment-based trial would be reinforcing to non-avoiders, it was less so for avoiders, potentially biasing them to reduce categorization attempts in the future and make avoidance responses instead on those trials. In fact, avoidance behavior was negatively correlated with *R0* (Fig 6D), so that subjects who valued *R0* most negatively tended to make the most avoidance responses.

It is important to note that the difference in *R0* value between avoiders and non-avoiders is one of degree, with both avoiders and non-avoiders tending to value it positively (Fig 5). Since *R0* is triggered by successful categorization on punishment-based trials, this fits the idea that avoiding a potential punishment is itself rewarding [26]. Of course, *R0* is also triggered by missed reward on reward-based trials; the fact that most subjects value *R0* as more like a reward than a punisher is consistent with the concept of loss aversion, which states that losses are psychologically more powerful than gains [27]. On the other hand, while this was true across subjects on average, Fig 6D shows that even among putatively healthy college undergraduates, there was considerable individual variation, with some subjects valuing *R0* negatively, i.e. as a punisher.

### Feedback on Avoided Trials

The present study also considered the interpretation of feedback when subjects were allowed to avoid trials by executing a “skip” response instead of attempting categorization. As shown in Fig 7, subjects who avoided more reward-based and punishment-based trials each tended to have larger estimated values of *RSrew* and *RSpun*, indicating they viewed this feedback as reinforcing. While it is not surprising that *RSpun* was, on average, valued more positively than *RSrew* (missed punishment is more reinforcing than missed reward, Fig 5), it is perhaps surprising that there were no significant differences whether this feedback took the form of neutral acknowledgment (in the no-feedback condition) vs. details about missed reward/punishment (in the feedback condition). Perhaps this simply indicates that subjects in the no-feedback condition who chose to avoid trials were perfectly aware of the potential consequences of their actions, and so provision of more explicit feedback was unnecessary and ineffective; alternately, it may be that subjects in the feedback condition did not process the feedback more deeply than they would have a simple acknowledgment. Further work might investigate this issue further, perhaps by including a forced-categorization test to determine whether subjects who were frequent avoiders nevertheless had learned the optimal category responses.

### Implications for understanding avoidance

One implication of this work is that absence of feedback is not necessarily the same as absence of reinforcement, and that task designs which include putatively neutral feedback cannot necessarily assume that such feedback is truly interpreted as neutral by the subjects. In fact, while the mathematically optimal solution to this task, which maximizes total points, is to avoid all punishment-based trials while attempting to categorize all reward-based trials, a large number of putatively-healthy, high-functioning subjects chose to approach the task differently. As noted above, this may be due to a preference for ambiguity or challenge over the “safe and boring” route of avoidance, combined with the fact that, for many subjects, successful categorization on punishment-based trials resulted in an outcome that was subjectively similar to explicit reward.

Our prior study in veterans [10] considered a version of the task where categorization was required on every trial; nevertheless, the findings from that study also indicated that veterans with few/no PTSD symptoms valued the no-feedback outcome as strongly positive. In contrast, those with severe PTSD symptoms valued it more neutrally. This had the curious effect of improving their performance on the reward-based trials, since for them there was a much larger distinction between explicit reward and *R0*. While, as demonstrated in Fig 6D of the current study, neutral- or negatively-valued *R0* can be observed even in putatively healthy subjects, one possibility is that this cognitive bias could be a mechanism which—while not necessarily pathological in itself—could contribute to development of PTSD and other anxiety disorders. For example, it was found that anxiety-vulnerable individuals have a similar negative bias, which is thought to result in a lower criterion for detecting danger and greater avoidance of ambiguous stimuli ("better safe than sorry" strategy) [28].

These data are also consistent with prior work examining emotional expressions in animals (and also humans) [29–31]. For example, Mendl et al. [31] argue that emotional states can be expressed in ambiguous situations, especially if the individual is living in a high-threat environment, which then lead to avoidance behavior. Future work should measure mood changes in PTSD individuals in both ambiguous and non-ambiguous situations, and test whether there are relationships between mood and avoidance behavior.

In the meantime, understanding how individuals use and learn from ambiguous and neutral feedback may have potential implications for the treatment of PTSD avoidance symptoms. Many studies have found that PTSD patients and exposure to trauma are associated with greater avoidance behavior, which is a core symptom of PTSD [32]. Our findings suggest that the provision of informational feedback may help reduce avoidance. Future work could examine this manipulation in individuals with PTSD, or in healthy adults with vulnerability factors that confer risk for anxiety or PTSD.

## Conclusions

The central purpose of this study was to better understand how subjects make decisions to avoid, and the role of ambiguous and informational feedback in this decision. In our prior study, we observed a surprising number of subjects who chose never to avoid, even though by doing so they would reduce their own risk of point loss (without increasing their chance for point gain). One possible interpretation is that the lack of feedback on avoidance trials produced an uncertainty which many subjects found aversive; in this case, providing informational feedback should increase avoidance. However, we found that providing informational feedback actually reduced avoidance. Another interpretation is that many subjects do not avoid because the absence of punishment on successfully-categorized punishment-based trials is itself reinforcing. This interpretation is consistent with modeling results that indicated higher values of *R0*, the value of ambiguous feedback, among subjects who did not make use of the avoidance response. These empirical and modeling results therefore emphasize the important role of ambiguous, neutral, and uncertain outcomes in avoidance behavior, and continue to suggest that variability in valuation of ambiguous outcomes is an important factor contributing to individual differences in avoidance.

## Supporting Information

S1 File This is the S1 file; it is a.zip file containing raw empirical data. It includes one.doc file per subject (199 files total), with output from the computer-based task described in the text. A “readme” file is included explaining how the output should be interpreted and providing basic demographic data for the subjects (ID, age, gender, experimental condition).
(DOCX)

S2 File This is the S2 file; it is a.docx file containing the C code used to implement the RL model described in the text. Information contained in the file header describes how to use and run the program.
(ZIP)
