# Supplementary material for: The Role of Informative and Ambiguous Feedback in Avoidance Behavior: Empirical and Computational Findings
Source: PLoS One. 2015 Dec 2;10(12):e0144083. doi: 10.1371/journal.pone.0144083 (PMC4668119; doi:10.1371/journal.pone.0144083)
Supplement: S1 File — It includes one.doc file per subject (199 files total), with output from the computer-based task described in the text. A “readme” file is included explaining how the output should be interpreted and providing basic demographic data for the subjects (ID, age, gender, experimental condition). (DOCX) [file pone.0144083.s001.docx]

/*

RL_code.txt:

This code implements the RL model described in the accompanying text (Moustafa et al., 2015/under review).

The code was written by Catherine E. Myers. Development and testing of this software was funded by the Department of Veterans Affairs, Office of Research and Development, Clinical Science Research and Development. This software is in the public domain, and may be freely copied and used in non-commercial products, providing that the following statement is included: “This software [is adapted from software which] was written by Catherine E. Myers, under funding from the Department of Veterans Affairs, Office of Research and Development, as originally published in Moustafa et al., 2015/under review.” No guarantees are given or implied. Use of this program implies acceptance of these terms.

Instructions for running code:

1. The program was written in C to run under the Mac OS X Xcode (version 5.1.1) environment; however, it was written to make minimal use of library functions so as to be fairly easy to compile/run under any C compiler.

2. This version of the software implements the six-parameter model, including free parameters LR+ (here, LRP), LR- (here, LRP), T (here, TEMP), R0, RSrew, and RSpun. To implement simpler (nested) models, set search space for unused parameters to 0 or another fixed value as appropriate.

3. When user is queried for “task” version: 7=”standard” version of the task (no skipping); 8=”no-feedback” version of the task (skipping response allowed), 9=”feedback” version of the task (skipping allowed and results in informational feedback).

4. The code expects as input a data file for each subject, as generated from the “quarters” task software. (For description of task methodology, see Moustafa et al., 2015/under review.) These should be <name>.doc files where <name> is the subject ID, and contain 15 header lines (stripped for privacy in the documents provided as supplemental material to Moustafa et al.), then one line per trial of the form “n s r c o RT t” where n=trial number, s=<stimulus name, stimulus ID, correct response, points won for correct response, points lost for incorrect response>, r=subject response (A, B, or s), c=subject’s response was correct (1) or incorrect (0) in the sense of generating point gain (or avoiding point loss), o=subject’s response was optimal (1) or non-optimal (0) for this stimulus regardless of points gained/lost on this trial, t=reaction time (RT) in system ticks. Items c and o are suppressed if the subject skipped the trial. The program conducts grid search on the free parameters and outputs the combination of parameter values which best fit that data, in terms of minimal LLE. Each search is conducted both forward and backward; if identical numbers are identified each time, then a unique configuration of “best-fit” parameter values generates minimal LLE.

5. Output appears in the console window and can be redirected to a .txt file for later analysis.

6. Search is conducted both “forward” and “backward” through the search space; if the same values are returned both ways, a unique configuration of “best-fit” parameters has been identified. Otherwise, multiple parameter configurations may return minimal LLE.

*/

#import <Cocoa/Cocoa.h>

#include <stdio.h>

#include <stdlib.h>

#define MAX_TRIALS 160

#define FIRST_TRIAL 0

#define NUM_STIM 4

#define CATEGORY_A 0

#define CATEGORY_B 1

#define SKIP 2

#define NUM_RESP 3

#define Q_INIT 0.0 // initialize the Qs to this value

// Free parameters, with min/max value for each, and stepsize for grid search of parameter space

// Note: for simpler models, can set min=max to hold a particular parameter constant

// LRG = learning rate when PE>0 (defined as “LR+” in text)

#define LRGmin 0.0

#define LRGmax 1.0

#define LRGstepsize 0.05

// LRP = learning rate when PE<0 (defined as “LR-” in text)

#define LRPmin 0.0

#define LRPmax 1.0

#define LRPstepsize 0.05

// TEMP = Temperature: low=exploit (choose response with highest Q) vs. high=explore

// (randomly choose response) (defined as “T” in text)

#define TEMPmin 0.0

#define TEMPmax 1.0

#define TEMPstepsize 0.05

// R0 = reinforcement value of R0 (no-feedback outcome)

#define R0min -1.0

#define R0max 1.0

#define R0stepsize 0.1

// RSAB = reinforcement value of RS (skipping feedback) on reward-based trials (stimuli A&B)

#define RSABmin -1.0

#define RSABmax 1.0

#define RSABstepsize 0.1

// RSCD = reinforcement value of RS (skipping feedback) on punishment-based trials (stimuli C&D)

#define RSCDmin -1.0

#define RSCDmax 1.0

#define RSCDstepsize 0.1

int Q_TASK; // task v. 7 (no skipping), or 8 or 9

float Q[NUM_RESP][NUM_STIM]; // weight for each response to each stimulus

float Pa[NUM_RESP][MAX_TRIALS]; // probability of choosing response r on each trial

// main():read in a stripped file, do grid search, and return the parameters generating lowest

// LLE for that subject’s data; loop until filename “x” is received.

int main()

{

FILE *fp;

char stimulus[MAX_TRIALS], filename[200], pathname[200], shortname[100];

int p, x, t, response[MAX_TRIALS], outcome[MAX_TRIALS], skip_trials=0;

int optRew, optPun;

int rewSkip, punSkip;

float LRG, LRP, TEMP, R0, RSAB, RSCD; // free parameters to optimize

float LRGopt, LRPopt, TEMPopt, R0opt, RSABopt, RSCDopt; // current "best-fit" values

float my_outcome;

double curMaxLLE;

double LLE, temp;

int run_model();

extern float Q[NUM_RESP][NUM_STIM];

extern float Pa[NUM_RSP][MAX_TRIALS];

char c1, c2;

(void) srandom( time (NULL) ); // seed the random number generator off the system clock

if (FIRST_TRIAL!=0) printf("NOTE: STARTING FROM TRIAL %d\n", FIRST_TRIAL);

if (MAX_TRIALS!=160) printf("NOTE: ONLY CALCULATING TO TRIAL %d\n", MAX_TRIALS);

printf("task version: "); scanf("%d", &Q_TASK);

if ((Q_TASK!=7)&&(Q_TASK!=8)&&(Q_TASK!=9)) { printf("Unknown task.\n"); exit(0); }

// first, read in subject's trial order, responses, and feedback

printf("\ninput path name (e.g. /Users/..../");

scanf("%s", pathname); // directory in which the input files are found, starting from root

p=0; while (pathname[p]!='\0') { filename[p]=pathname[p]; p++; }

printf("\ninput file name (e.g. 001.doc): ");

scanf("%s", shortname); // individual file names in the directory (ending in .doc)

while (shortname[0]!='x') {

x=0;

while (shortname[x]!='\0') { filename[p+x]=shortname[x]; x++; } filename[p+x]='\0';

fp=fopen(filename,"r");

if (fp==NULL) {

printf("can't open file %s\n", filename);

exit(EXIT_FAILURE);

}

else { // process this file

printf("File %s opened successfully.\n", filename);

// Strip off header

while ((c1=getc(fp))!='?') ; while ((c1=getc(fp))!='?') ;

for (t=0, optRew=0, optPun=0, rewSkip=0, punSkip=0;t<MAX_TRIALS;t++) {

fscanf(fp, "%*d %*c%*c%*c%*c%*c%*cPict,Stim%c,%*c,%*d,%*d %c", &c1, &c2);

stimulus[t]=c1;

if ((c2=='A')||(c2=='B')) { // categorization response

response[t]=c2-'A'; // convert to A=0, B=1

fscanf(fp, "%d %*d %*s %*d", &x);

if ((x==1)&&((c1=='A')||(c1=='B'))) outcome[t]=1;

else if ((x==0)&&((c1=='C')||(c1=='D'))) outcome[t]= -1;

else outcome[t]=0;

}

else { // skip response

response[t]=2; // convert to skip=2

fscanf(fp, "%*s %*d\n");

if (Q_TASK==9) { // skipping response generates feedback

if ((c1=='A')||(c1=='B')) outcome[t]=1;

else outcome[t]= -1;

}

else outcome[t]=0; // skipping response generates no feedback

}

// finally, just count optimal reward and punish trials for this subject

if (stimulus[t]=='A') {

if (response[t]==0) optRew++;

else if (response[t]==2) rewSkip++;

}

else if (stimulus[t]=='B') {

if (response[t]==1) optRew++;

else if (response[t]==2) rewSkip++;

}

else if (stimulus[t]=='C') {

if (response[t]==0) optPun++;

else if (response[t]==2) punSkip++;

}

else {

if (response[t]==1) optPun++;

else if (response[t]==2) punSkip++;

}

}

fclose(fp);

} // end process this file

// SEARCH GRID SPACE, FORWARD

// initialize optimal parms

curMaxLLE= -999999.0;

LRGopt=LRGmin; LRPopt=LRPmin; TEMPopt=TEMPmin; R0opt=R0min; RSABopt=RSABmin; RSCDopt=RSCDmin;

// next, for all possible parameters, run model on that trial order

for (LRG=LRGmin; LRG<=LRGmax+0.001; LRG+=LRGstepsize)

for (LRP=LRPmin; LRP<=LRPmax+0.001; LRP+=LRPstepsize)

for (TEMP=TEMPmin; TEMP<=TEMPmax+0.001; TEMP+=TEMPstepsize)

for (R0=R0min; R0<=R0max+0.001; R0+=R0stepsize)

for (RSAB=RSABmin; RSAB<=RSABmax+0.001; RSAB+=RSABstepsize)

for (RSCD=RSCDmin; RSCD<=RSCDmax+0.001; RSCD+=RSCDstepsize)

{ // for current combination of parameter values...

// initialize the model

for (t=0;t<NUM_STIM;t++)

for (x=0;x<NUM_RESP;x++) {

Q[x][t]=Q_INIT;

}

// run model: for each trial, record prob Pa of generating response "A"

skip_trials=0;

for (t=FIRST_TRIAL;t<MAX_TRIALS;t++) {

if (response[t]==SKIP) {

if ((stimulus[t]=='A')||(stimulus[t]=='B'))

my_outcome=RSAB; else my_outcome=RSCD;

skip_trials++;

}

else if (outcome[t]==0) {

my_outcome=R0;

}

else my_outcome=1.0*outcome[t];

(void) run_model(t, LRG, LRP, TEMP, 0.0, stimulus[t]-'A',

response[t], my_outcome);

} // end for all trials

// calc LLE: for all trials t, prob model makes same response as subject

LLE=0.0;

for (t=FIRST_TRIAL;t<MAX_TRIALS;t++) {

temp=(double) Pa[response[t]][t];

if (temp==0.0) temp=0.001; // log(0)--> inf

LLE+=(float)log(temp); // LLE += P(modelR=subjectR) on trial t

}

// if this is best LLE so far, save parm values as new "optimal values"

if (LLE > curMaxLLE) {

LRGopt=LRG; LRPopt=LRP; TEMPopt=TEMP; R0opt=R0;

RSABopt=RSAB; RSCDopt=RSCD; curMaxLLE=LLE;

}

} // ... end for current combination of parameter values

printf("%s ", shortname);

printf("RewOpt %4.2f PunOpt %4.2f ", (100.0*optRew)/((0.5*MAX_TRIALS)-(1.0*rewSkip)),

(100.0*optPun)/((0.5*MAX_TRIALS)-(1.0*punSkip)));

if (Q_TASK>7) printf("rewSkips %d punSkips %d ", rewSkip, punSkip);

printf(" LRG %4.2f LRP %4.2f TEMP %4.2f R0 %4.2f ", LRGopt, LRPopt, TEMPopt, R0opt);

if (Q_TASK>7) printf("RSAB %4.2f RSCD %4.2f ",RSABopt, RSCDopt);

printf("LLE %4.2f ", curMaxLLE);

// SEARCH GRID SPACE, BACKWARD

// initialize optimal parms

curMaxLLE= -999999.0;

LRGopt=LRGmax; LRPopt=LRPmax; TEMPopt=TEMPmax; R0opt=R0max; RSABopt=RSABmax; RSCDopt=RSCDmax;

// next, search in reverse order, just to make sure no duplicate "best-fit" solutions

for (LRG=LRGmax; LRG>=LRGmin-0.001; LRG-=LRGstepsize)

for (LRP=LRPmax; LRP>=LRPmin-0.001; LRP-=LRPstepsize)

for (TEMP=TEMPmax; TEMP>=TEMPmin-0.001; TEMP-=TEMPstepsize)

for (R0=R0max; R0>=R0min-0.001; R0-=R0stepsize)

for (RSAB=RSABmax; RSAB>=RSABmin-0.001; RSAB-=RSABstepsize)

for (RSCD=RSCDmax; RSCD>=RSCDmin-0.001; RSCD-=RSCDstepsize)

{ // for current combination of parameter values...

// initialize the model

for (t=0;t<NUM_STIM;t++)

for (x=0;x<NUM_RESP;x++) {

Q[x][t]=Q_INIT;

}

// run model: for each trial, record prob of response "A"

skip_trials=0;

for (t=FIRST_TRIAL;t<MAX_TRIALS;t++) {

if (response[t]==SKIP) {

if ((stimulus[t]=='A')||(stimulus[t]=='B')) my_outcome=RSAB;

else my_outcome=RSCD;

skip_trials++;

}

else if (outcome[t]==0) {

my_outcome=R0;

}

else my_outcome=1.0*outcome[t];

(void) run_model(t, LRG, LRP, TEMP, 0.0, stimulus[t]-'A',

response[t], my_outcome);

}

// calc LLE: for all trials t, prob model makes same response as subject

LLE=0.0;

for (t=FIRST_TRIAL;t<MAX_TRIALS;t++) {

temp=(double) Pa[response[t]][t];

if (temp==0.0) temp=0.001; // log(0)--> inf

LLE+=(float)log(temp); // LLE += P(modelR=subjectR) on trial t

}

// if this is best LLE so far, save parm values as new "optimal values"

if (LLE > curMaxLLE) {

LRGopt=LRG; LRPopt=LRP; TEMPopt=TEMP; R0opt=R0;

RSABopt=RSAB; RSCDopt=RSCD; curMaxLLE=LLE;

}

} // ... end for current combination of parameter values

printf("BACK ");

printf("%4.2f %4.2f %4.2f %4.2f ", LRGopt, LRPopt, TEMPopt, R0opt);

if (Q_TASK>7) printf("%4.2f %4.2f ",RSABopt, RSCDopt);

printf("%4.2f ", curMaxLLE);

printf("\n");

// read in next filename

printf("\ninput next file name (.doc): ");

scanf("%s", shortname);

} // end while

return TRUE;

}

// Run the model: first, calc probability Prob of choosing response "A" on the current trial;

// then update V for the current stimulus and (actual) response based on the obtained outcome.

// This is the GAIN-LOSS model from Frank 2007 PNAS updated as in Moustafa et al. (under review).

int run_model(t, LRG, LRP, TEMP, PERSEV, stimulus, response, my_outcome)

int t;

float LRG, LRP, TEMP, PERSEV;

int stimulus, response;

float my_outcome;

{

extern float Q[NUM_RESP][NUM_STIM];

extern float Pa[NUM_RESP][MAX_TRIALS];

float eA, eB, eSKIP, denom, PE;

//NB calculate ProbA before updating the Vs!

eA=exp(Q[CATEGORY_A][stimulus]/TEMP);

eB=exp(Q[CATEGORY_B][stimulus]/TEMP);

if (Q_TASK<8) { // version 7 does not allow skip response

denom=eA+eB;

Pa[CATEGORY_A][t]=eA/denom;

Pa[CATEGORY_B][t]=eB/denom;

}

else {

eSKIP=exp(Q[SKIP][stimulus]/TEMP);

denom=eA+eB+eSKIP;

Pa[CATEGORY_A][t]=eA/denom;

Pa[CATEGORY_B][t]=eB/denom;

Pa[SKIP][t]=eSKIP/denom;

}

PE=my_outcome-Q[response][stimulus];

if (PE>0.0) // change for the better (gain)

Q[response][stimulus]+=LRG*PE;

else if (PE<0.0) // change for the worse (loss)

Q[response][stimulus]+=LRP*PE;

else ; // NB no change if PE=0

return(TRUE);

}
