# Supplementary material for: The Role of Informative and Ambiguous Feedback in Avoidance Behavior: Empirical and Computational Findings
Source: PLoS One. 2015 Dec 2;10(12):e0144083. doi: 10.1371/journal.pone.0144083 (PMC4668119; doi:10.1371/journal.pone.0144083)
Supplement: S2 File — Information contained in the file header describes how to use and run the program. (ZIP) [file pone.0144083.s002.zip › Moustafa2015_data/README.docx]

The data in these folders (Moustafa2015_NoFeedback/001-100 and Moustafa2015_Feedback/001-100) are de-identified versions of raw data of Moustafa et al. (2015), no-feedback and feedback conditions respectively. See article for full methodological details.

These data were collected under a human subjects protocol approved by the Rutgers University IRB (protocol #04-003M15; PI=Catherine Myers); data collection and analysis were partially funded by the NSF/NIH Collaborative Research in Computational Neuroscience (CRCNS) Program and NIAAA R01 AA018737, and by Award Number I01 CX000771 from the Clinical Science Research and Development Service of the VA Office of Research and Development. These data are in the public domain for non-commercial use. Any work using or citing these data should acknowledge both the original publications and the funding agencies.

Each subject’s data are in one file, with filenames corresponding to subject ID number. (Raw data files generated by the computer task contain additional information, including testing data and experimenter name, which have been stripped here for privacy.) In the file, each row is one trial, of the format:

```
1 Worms_Pict,StimA,B,25,0   B 1  0    RT 263
2 Green_Pict,StimB,B,25,0   A 0  0    RT 186
3 SticksPict,StimC,A,0,25  s    RT 109
```

Etc.

First item is trial number (1, 2, … 160).

Second item contains five components:

- Which picture was shown on that trial (Worms_Pict, Circle_Pict, Green_Pict, Sticks_Pict); note these pictures are randomly mapped to stimulus ID (Stimuli A & B are used on reward-based trials; Stimuli C & D are used on punishment-based trials) and class (Stimuli A& C belong to category A on 80% of trials and to category B on 20% of trials; Stimuli B & D belong to category B on 80% of trials and to category A on 20% of trials); specific mappings for each subject appear in the header.
- Stimulus ID
- Category to which the stimulus belongs on the current trial (category A or B)
- Possible reward if subject responds correctly (25 on reward-based trials; 0 on punishment-based trials)
- Possible punishment if subject responds incorrectly (0 on reward-based trials; 25 on punishment-based trials).

Third item is subject’s actual response (A, B, or s=skip). Fourth item is 1 if subject obtained reward (or avoided punishment) and 0 if subject did not; note this is blank if response was skip. Fifth item is 1 if subject’s categorization response was the optimal (most-often rewarded or non-punished) response for that subject, regardless of the actual outcome on this trial; note this is blank if response was skip. Sixth/seventh item is RT (reaction time).

Demographic information for the subjects appears in the accompanying file Moustafa2015_demog.xls.
