# Supplementary material for: The Role of Informative and Ambiguous Feedback in Avoidance Behavior: Empirical and Computational Findings
Source: PLoS One. 2015 Dec 2;10(12):e0144083. doi: 10.1371/journal.pone.0144083 (PMC4668119; doi:10.1371/journal.pone.0144083)
Supplement: S2 File — Information contained in the file header describes how to use and run the program. (ZIP) [file pone.0144083.s002.zip › Moustafa2015_data/Moustafa2015_NoFeedback/100.doc]

1 CirclePict,StimD,A,0,25	B 0 1	RT 310

2 Worms_Pict,StimC,A,0,25	A 1 1	RT 85

3 SticksPict,StimA,A,25,0	A 1 1	RT 44

4 CirclePict,StimD,B,0,25	B 1 1	RT 28

5 Green_Pict,StimB,B,25,0	A 0 0	RT 18

6 SticksPict,StimA,A,25,0	B 0 0	RT 33

7 CirclePict,StimD,B,0,25	A 0 0	RT 24

8 Worms_Pict,StimC,A,0,25	A 1 1	RT 32

9 SticksPict,StimA,B,25,0	B 1 0	RT 21

10 CirclePict,StimD,B,0,25	A 0 0	RT 20

11 CirclePict,StimD,B,0,25	B 1 1	RT 54

12 SticksPict,StimA,A,25,0	B 0 0	RT 16

13 Green_Pict,StimB,B,25,0	A 0 0	RT 11

14 SticksPict,StimA,B,25,0	B 1 0	RT 14

15 Green_Pict,StimB,B,25,0	B 1 1	RT 17

16 Worms_Pict,StimC,A,0,25	A 1 1	RT 13

17 Worms_Pict,StimC,A,0,25	A 1 1	RT 81

18 CirclePict,StimD,B,0,25	B 1 1	RT 75

19 SticksPict,StimA,A,25,0	B 0 0	RT 86

20 Worms_Pict,StimC,A,0,25	A 1 1	RT 38

21 SticksPict,StimA,A,25,0	B 0 0	RT 51

22 Green_Pict,StimB,B,25,0	B 1 1	RT 61

23 SticksPict,StimA,A,25,0	B 0 0	RT 65

24 SticksPict,StimA,A,25,0	B 0 0	RT 52

25 Worms_Pict,StimC,B,0,25	A 0 1	RT 51

26 Worms_Pict,StimC,A,0,25	A 1 1	RT 43

27 Worms_Pict,StimC,B,0,25	A 0 1	RT 37

28 CirclePict,StimD,B,0,25	A 0 0	RT 43

29 Green_Pict,StimB,A,25,0	B 0 1	RT 27

30 Green_Pict,StimB,A,25,0	B 0 1	RT 17

31 CirclePict,StimD,B,0,25	B 1 1	RT 35

32 Green_Pict,StimB,B,25,0	A 0 0	RT 18

33 SticksPict,StimA,A,25,0	B 0 0	RT 65

34 Worms_Pict,StimC,A,0,25	A 1 1	RT 35

35 Green_Pict,StimB,B,25,0	B 1 1	RT 76

36 CirclePict,StimD,A,0,25	A 1 0	RT 56

37 Green_Pict,StimB,B,25,0	B 1 1	RT 32

38 CirclePict,StimD,B,0,25	A 0 0	RT 87

39 Worms_Pict,StimC,A,0,25	A 1 1	RT 60

40 Green_Pict,StimB,B,25,0	B 1 1	RT 39

41 SticksPict,StimA,A,25,0	A 1 1	RT 48

42 Worms_Pict,StimC,A,0,25	B 0 0	RT 62

43 Worms_Pict,StimC,A,0,25	A 1 1	RT 43

44 CirclePict,StimD,A,0,25	A 1 0	RT 60

45 Green_Pict,StimB,B,25,0	B 1 1	RT 58

46 Green_Pict,StimB,B,25,0	B 1 1	RT 33

47 SticksPict,StimA,B,25,0	A 0 1	RT 58

48 SticksPict,StimA,A,25,0	A 1 1	RT 29

49 CirclePict,StimD,A,0,25	A 1 0	RT 44

50 Worms_Pict,StimC,A,0,25	B 0 0	RT 55

51 Green_Pict,StimB,A,25,0	B 0 1	RT 38

52 Green_Pict,StimB,B,25,0	B 1 1	RT 63

53 Worms_Pict,StimC,B,0,25	B 1 0	RT 33

54 Green_Pict,StimB,B,25,0	B 1 1	RT 38

55 CirclePict,StimD,B,0,25	A 0 0	RT 45

56 Worms_Pict,StimC,B,0,25	B 1 0	RT 23

57 Green_Pict,StimB,B,25,0	B 1 1	RT 44

58 Green_Pict,StimB,B,25,0	B 1 1	RT 26

59 Worms_Pict,StimC,A,0,25	A 1 1	RT 41

60 CirclePict,StimD,B,0,25	A 0 0	RT 110

61 Worms_Pict,StimC,A,0,25	A 1 1	RT 56

62 Green_Pict,StimB,A,25,0	B 0 1	RT 31

63 Green_Pict,StimB,B,25,0	B 1 1	RT 25

64 CirclePict,StimD,B,0,25	B 1 1	RT 20

65 Worms_Pict,StimC,A,0,25	A 1 1	RT 47

66 CirclePict,StimD,B,0,25	A 0 0	RT 68

67 SticksPict,StimA,B,25,0	A 0 1	RT 59

68 Worms_Pict,StimC,A,0,25	A 1 1	RT 51

69 SticksPict,StimA,A,25,0	A 1 1	RT 43

70 SticksPict,StimA,A,25,0	A 1 1	RT 29

71 CirclePict,StimD,B,0,25	B 1 1	RT 46

72 Worms_Pict,StimC,A,0,25	A 1 1	RT 41

73 CirclePict,StimD,B,0,25	B 1 1	RT 31

74 SticksPict,StimA,A,25,0	A 1 1	RT 43

75 SticksPict,StimA,A,25,0	A 1 1	RT 43

76 SticksPict,StimA,A,25,0	A 1 1	RT 29

77 Green_Pict,StimB,B,25,0	A 0 0	RT 37

78 CirclePict,StimD,B,0,25	B 1 1	RT 55

79 SticksPict,StimA,A,25,0	A 1 1	RT 37

80 CirclePict,StimD,B,0,25	B 1 1	RT 53

81 Green_Pict,StimB,B,25,0	B 1 1	RT 53

82 Worms_Pict,StimC,A,0,25	A 1 1	RT 41

83 CirclePict,StimD,A,0,25	B 0 1	RT 45

84 Worms_Pict,StimC,A,0,25	A 1 1	RT 41

85 Green_Pict,StimB,A,25,0	B 0 1	RT 42

86 SticksPict,StimA,A,25,0	A 1 1	RT 47

87 SticksPict,StimA,A,25,0	A 1 1	RT 55

88 Green_Pict,StimB,B,25,0	B 1 1	RT 42

89 Worms_Pict,StimC,A,0,25	A 1 1	RT 85

90 Green_Pict,StimB,B,25,0	B 1 1	RT 41

91 CirclePict,StimD,B,0,25	B 1 1	RT 77

92 CirclePict,StimD,B,0,25	B 1 1	RT 58

93 Worms_Pict,StimC,B,0,25	A 0 1	RT 44

94 SticksPict,StimA,A,25,0	A 1 1	RT 40

95 Worms_Pict,StimC,A,0,25	A 1 1	RT 44

96 Worms_Pict,StimC,B,0,25	A 0 1	RT 45

97 SticksPict,StimA,B,25,0	A 0 1	RT 56

98 Green_Pict,StimB,B,25,0	B 1 1	RT 37

99 Green_Pict,StimB,B,25,0	B 1 1	RT 26

100 Green_Pict,StimB,B,25,0	B 1 1	RT 31

101 Green_Pict,StimB,A,25,0	B 0 1	RT 40

102 SticksPict,StimA,A,25,0	A 1 1	RT 39

103 SticksPict,StimA,A,25,0	A 1 1	RT 40

104 CirclePict,StimD,B,0,25	B 1 1	RT 93

105 Worms_Pict,StimC,A,0,25	A 1 1	RT 57

106 Green_Pict,StimB,B,25,0	B 1 1	RT 37

107 Worms_Pict,StimC,A,0,25	A 1 1	RT 53

108 CirclePict,StimD,B,0,25	B 1 1	RT 56

109 SticksPict,StimA,A,25,0	A 1 1	RT 39

110 Worms_Pict,StimC,A,0,25	A 1 1	RT 54

111 CirclePict,StimD,A,0,25	B 0 1	RT 50

112 CirclePict,StimD,B,0,25	B 1 1	RT 103

113 SticksPict,StimA,B,25,0	A 0 1	RT 36

114 CirclePict,StimD,B,0,25	B 1 1	RT 49

115 CirclePict,StimD,B,0,25	B 1 1	RT 43

116 SticksPict,StimA,A,25,0	A 1 1	RT 39

117 CirclePict,StimD,B,0,25	A 0 0	RT 48

118 Green_Pict,StimB,B,25,0	B 1 1	RT 29

119 SticksPict,StimA,A,25,0	A 1 1	RT 41

120 Worms_Pict,StimC,A,0,25	A 1 1	RT 51

121 Worms_Pict,StimC,A,0,25	B 0 0	RT 89

122 Worms_Pict,StimC,A,0,25	A 1 1	RT 43

123 Green_Pict,StimB,B,25,0	B 1 1	RT 37

124 SticksPict,StimA,B,25,0	A 0 1	RT 49

125 Green_Pict,StimB,B,25,0	B 1 1	RT 49

126 Green_Pict,StimB,B,25,0	B 1 1	RT 29

127 SticksPict,StimA,A,25,0	A 1 1	RT 40

128 SticksPict,StimA,A,25,0	A 1 1	RT 64

129 CirclePict,StimD,B,0,25	B 1 1	RT 44

130 Green_Pict,StimB,B,25,0	B 1 1	RT 41

131 Green_Pict,StimB,A,25,0	B 0 1	RT 29

132 Worms_Pict,StimC,A,0,25	A 1 1	RT 43

133 CirclePict,StimD,B,0,25	B 1 1	RT 51

134 CirclePict,StimD,A,0,25	A 1 0	RT 41

135 CirclePict,StimD,B,0,25	B 1 1	RT 45

136 CirclePict,StimD,B,0,25	B 1 1	RT 65

137 SticksPict,StimA,A,25,0	A 1 1	RT 70

138 SticksPict,StimA,A,25,0	A 1 1	RT 46

139 SticksPict,StimA,A,25,0	A 1 1	RT 48

140 Worms_Pict,StimC,A,0,25	A 1 1	RT 70

141 SticksPict,StimA,A,25,0	A 1 1	RT 87

142 Green_Pict,StimB,B,25,0	B 1 1	RT 51

143 Green_Pict,StimB,B,25,0	B 1 1	RT 70

144 CirclePict,StimD,B,0,25	A 0 0	RT 80

145 SticksPict,StimA,B,25,0	A 0 1	RT 46

146 Worms_Pict,StimC,A,0,25	A 1 1	RT 36

147 CirclePict,StimD,B,0,25	B 1 1	RT 69

148 SticksPict,StimA,A,25,0	A 1 1	RT 35

149 Worms_Pict,StimC,A,0,25	A 1 1	RT 66

150 CirclePict,StimD,B,0,25	B 1 1	RT 87

151 Green_Pict,StimB,B,25,0	B 1 1	RT 106

152 Green_Pict,StimB,A,25,0	B 0 1	RT 77

153 CirclePict,StimD,B,0,25	B 1 1	RT 71

154 Worms_Pict,StimC,A,0,25	A 1 1	RT 54

155 CirclePict,StimD,A,0,25	B 0 1	RT 96

156 Worms_Pict,StimC,B,0,25	A 0 1	RT 43

157 Green_Pict,StimB,B,25,0	B 1 1	RT 41

158 Worms_Pict,StimC,B,0,25	A 0 1	RT 56

159 Worms_Pict,StimC,A,0,25	A 1 1	RT 59

160 SticksPict,StimA,A,25,0	A 1 1	RT 80
